# Lattice Distortion in In_3_SbTe_2_ Phase Change Material with Substitutional Bi

**DOI:** 10.1038/srep12867

**Published:** 2015-08-11

**Authors:** Minho Choi, Heechae Choi, Seungchul Kim, Jinho Ahn, Yong Tae Kim

**Affiliations:** 1 Hanyang University, Department of Materials Science and Engineering, Seoul 133-791, Korea; 2 Korea Institute of Science and Technology, Center for Computational Science, Seoul 136-791, Korea; 3 Korea Institute of Science and Technology, Semiconductor Materials and Device Laboratory, Seoul 136-791, Korea

## Abstract

Sb atoms in In_3_SbTe_2_ (IST) are partially substituted by 3.2–5.5 at.% of Bi atoms. As a result, the NaCl crystal structure of IST is slightly distorted. The distorted inter-planar angles observed with fast Fourier transformation of the lattice images are within the maximum range of interplanar angles calculated by density functional theory. When the Bi content is increased, the crystallization temperature becomes relatively lower than that of IST, the activation energy decreases from 5.29 to 2.61 eV, and the specific heat and melting point are obviously reduced. Consequently, phase change random access memory (PRAM) fabricated with Bi-doped IST (Bi-IST) can operate with lower power consumption than pure IST PRAM. The set and reset speeds of PRAM cells fabricated with Bi-IST are both 100 ns with 5.5 at.% Bi, which are obviously faster than the switching speeds of PRAM cells fabricated with IST and Ge_2_Sb_2_Te_5_ (GST). These experimental results reveal that the switching speed is closely related with the thermal properties of the distorted lattice structure.

Phase change random access memory (PRAM) is the best one of candidates for next-generation universal memory. Phase change material, known as chalcogenide, is a core material to divide resistances according to phase change by electrical pulse converted into joule heating. Phase change alloys have been intensively investigated for PRAM applications because of their reversible switching between the amorphous and crystalline states, resulting in variable resistance levels^1^,^2^,^3^,^4^,^5^. This universal storage could replace existing data storage (non-volatile memory, CD&DVD disk, and hard disk), further, develop new fields (storage class memory and neuromorphic system). However, a number of switching operation, repeated by electric pulses, would induce decoding error with gradual change of resistance. The resistance drift within the narrow resistance difference leads to random fluctuations in the programmed levels^6^,^7^,^8^. Among several phase change alloys, In-Sb-Te alloys have been suggested as promising candidates for multi-level PRAM because IST alloys demonstrate a stable multi-phase change mechanism from amorphous to NaCl-type cubic, leading to multiple resistance levels^9^,^10^,^11^. However, the thermal stability of IST may not correspond with the higher switching speed because of the energy required. The role of vacancies for GST alloys has been investigated, verifying that switching occurs between the metastable NaCl state and the amorphous state, and those phase transitions can be formed with low activation energies^12^,^13^,^14^,^15^ at the short timescales due to the vacancies^16^. The structural features of GST alloys allow local lattice distortions and reduce the activation energy. Similarly, if a certain impurity atom is added into IST, the relationship between the switching speed and lattice distortion can be confirmed. In this work, Bi atoms are selected as the impurity because Bi and Sb are part of the same group in the periodic table. The GST, IST, and IST with incorporated Bi atoms (Bi-IST) are investigated to identify the relationships between the atomic lattice structure, energy stability, activation energy, and switching speed.

## Results and Discussion

**Figure 1:**
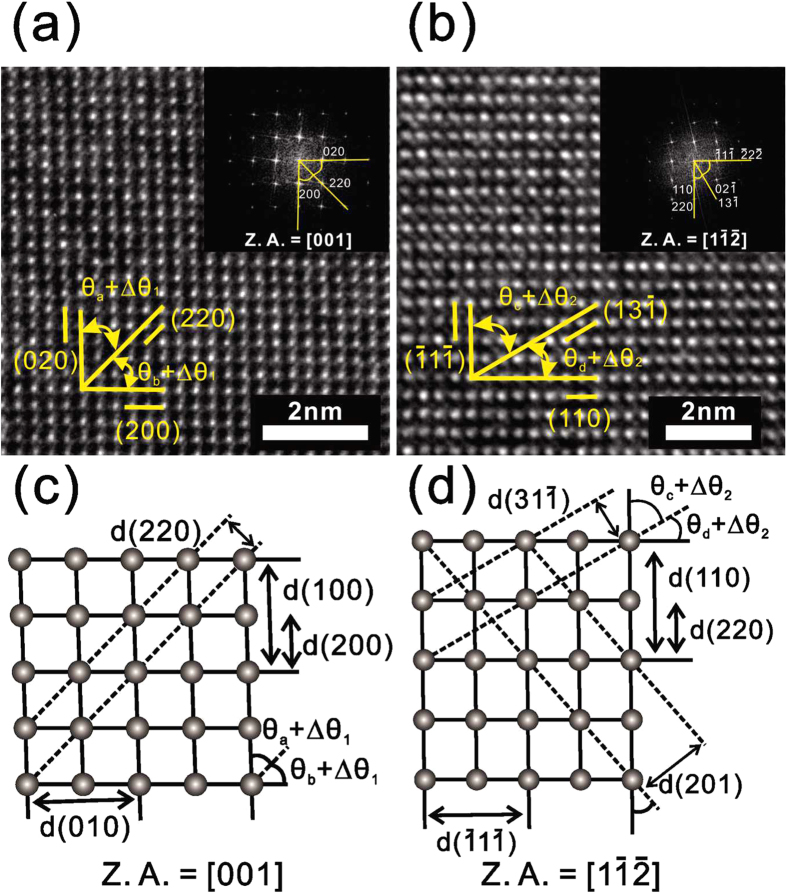
The HRTEM and FFT (inset) images of (**a**) 3.2 at.% Bi-IST represented along the [001] zone axis and (**b**) 5.5 at.% Bi-IST represented along the 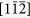 zone axis (θ_a,b,c,d_: 45°, 45°, 58.52°, 31.48°). Partially distorted lattice models of the NaCl-structured IST represented along (**c**) [001] and (**d**) 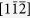 zone axes.

To confirm the effects of the substitutional Bi atoms on the IST alloy structure, the atomic lattice structures are thoroughly compared with pure IST. The high-resolution transmission electron microscopy (HRTEM) images of Bi-IST are obtained for samples with Bi contents of 3.2 at.% (Fig. 1(a)) and 5.5 at.% (Fig. 1(b)). The respective fast Fourier transform (FFT) images are inserted in Fig. 1(a,b). The atomic lattice models from these figures indicate that both Bi-IST samples have a NaCl structure along the [001] and 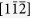 zone axes, as shown in Fig. 1(c,d), respectively. The interplanar angles are slightly distorted due to the presence of Bi atoms. The changed angle (Δθ) slightly increases with the addition of 3.2 and 5.5 at.% of Bi atoms, and the values are 0.84° < Δθ_1_ < 1.00° and 1.33° < Δθ_2_ < 2.06° along the [001] and 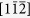 zone axes, respectively. The interplanar distances also slightly change with the distorted angles. However, the lattice structure is not seriously changed but partially distorted, and the lattice structure seems to be a bit more distorted as increasing the Bi contents.

According to previous results, Sb and Te atoms co-exist on the (1/4, 1/4, 1/4) sites in the zincblende structure of InSb when IST is transformed at the first crystallization state. These Sb and Te atoms move to (1/2, 1/2, 1/2) sites, forming rocksalt (NaCl) structured IST at the second phase transformation^10^,^11^. At the third phase changing state, In_3_SbTe_2_ separates into InTe and InSb, and only InTe appears in the crystallized phase. Meanwhile, the Bi atoms are expected to replace one atomic site among In, Sb, and Te, and the lattice structure becomes distorted because the atomic radius of Bi is different than that of the host atoms^17^.

**Figure 2:**
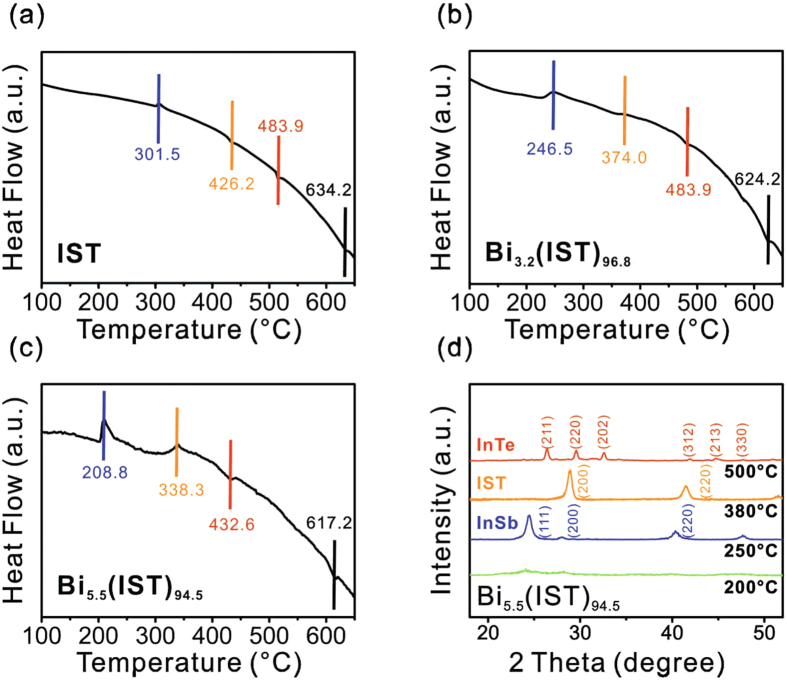
The DSC curves of (**a**) IST, (**b**) 3.2 at.% Bi-IST, and (**c**) 5.5at.% Bi-IST obtained with the heating rate of 10 °C/min. (**d**) The XRD patterns for 5.5 at.% Bi-IST annealed at 200, 250, 380, and 500 °C.

Figure 2(a) shows the differential scanning calorimetry (DSC) curve for IST, while Fig 2(b,c) show the DSC curves for the Bi-IST samples. The crystallization temperatures (T_c_) and the melting temperatures (T_m_) can be determined from these figures. As the Bi atom content increases from 0 to 5.5 at.%, the first transition temperature T_c1_ is reduced from 301.5 for IST to 246.5 and 208.8 °C for 3.2 at.% Bi and 5.5 at% Bi, respectively. The second and third transition temperatures are also clearly reduced by the increase of Bi atoms compared with that of IST, and the difference between the first and the third crystallization temperatures is also larger than that of IST. This difference is more significant than the previously obtained GST results because the first and second transition temperatures of GST are about 140 °C and 310 °C, respectively^18^. The expanded temperature ranges between phases enable more stable control over phase change from a certain phase to another phase and guarantee the resistance against the mixing with unwanted phase.

Figure 2 also shows that the melting temperature T_m_ of IST (634.2 °C) is reduced to 624.2 and 617.2 °C corresponding to the Bi contents of 3.2 and 5.5 at.%, respectively. Interestingly, for 5.5 at.% Bi, T_m_ (617.2 °C) is quite similar with T_m_ (616 °C) for GST^19^. The multi-level crystallization of Bi-IST (5.5 at.%) is also confirmed with X-ray diffractometry (XRD), as shown in Fig. 2(d). In this figure, the amorphous phase is maintained until 200 °C, and the sample crystallizes to the InSb phase at 250 °C, which is within the first transition temperature range. The InSbTe and InTe phases appear at 380 and 500 °C, respectively, corresponding with the second and third phase transition temperatures. However, Bi-IST clearly has the same NaCl structure as IST, meaning that the crystal structure is maintained but slightly distorted by the Bi atoms. Moreover, Bi-IST has the same sequence of multiple phase transitions from the amorphous phase to the InSb, InSbTe, and InTe phases^9^,^10^.

**Figure 3:**
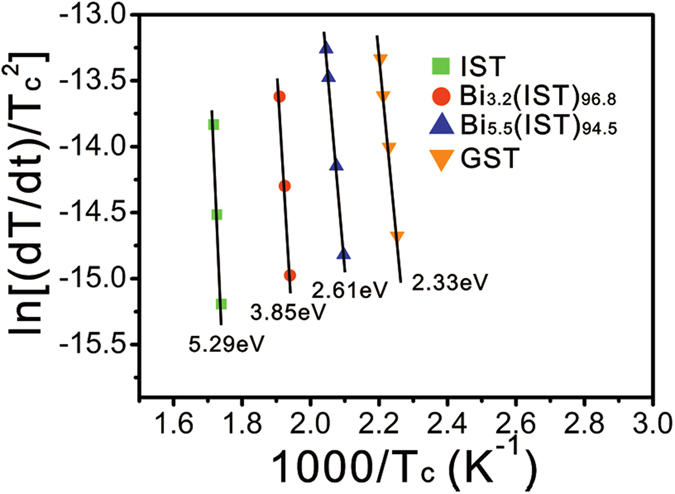
Kissinger’s plots and slopes for the activation energies of IST, Bi-IST alloys (3.2 and 5.5 at.% Bi), and GST.

From the non-isothermal DSC measurements, the activation energy is determined with the slope of Kissinger’s plot using


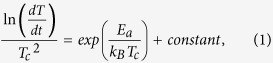


with *dT/dt* the heating rate, and *T*_*c*_ the crystallization temperature^20^. The activation energies for GST, IST, 3.2 at.% Bi-IST, and 5.5 at.% Bi-IST are 2.33, 5.29, 3.85, and 2.61 eV, respectively. A higher activation energy means that the material requires more energy to achieve phase transformation than other materials because the crystal growth rate corresponds to the activation energy for crystallization^21^. Figure 3 shows that the activation energy of GST is much lower than that of IST. Moreover, amorphous GST is easily transformed into the meta-stable face-centered cubic (FCC) structure. Therefore, the activation energy for GST is the lowest among these samples. However, higher activation energy indicates better thermal stability. Therefore, IST seems to be relatively better than GST, but this higher activation energy should be reduced. Adding Bi atoms into IST reduces the activation energy, as shown in Fig. 3. The reduced IST activation energy has an effect on decrease in barrier for phase change, and then atomic migration occurs more easily. The experimental results of Figs 2 and 3 indicate that the lower crystallization temperatures of Bi-IST are well matched with the lower activation energy, meaning that the phase transition occurs more easily with the addition of Bi atoms.

**Figure 4:**
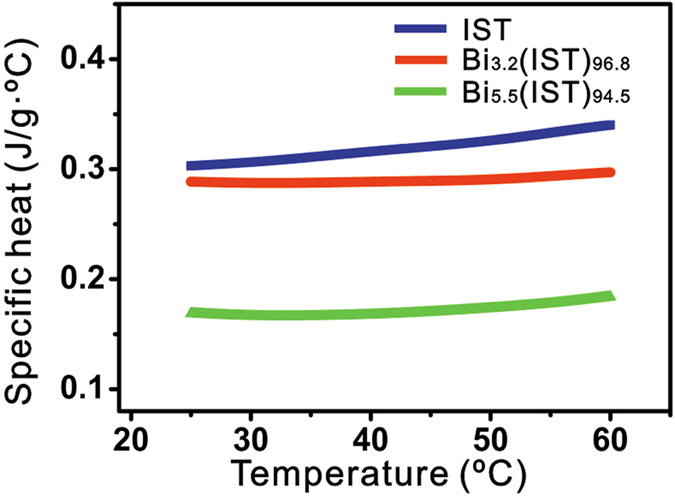
The specific heats of crystalline IST and Bi-IST obtained in the temperature range from 25 °C to 60 °C at a heating rate of 10 °C/min with constant pressure.

The specific heat of Bi-IST is investigated as a different effect of the distorted lattice structure. Figure 4 shows the measured specific heats (c_p_) for crystalline IST and for two different Bi-IST samples from room temperature (25 °C) to 60 °C. As the Bi concentration is increased, c_p_ is reduced from 0.302 < c_p,IST_ < 0.340 to 0.168 < c_p,5.5at.%Bi_ < 0.184. Consequently, the decrease in c_p_ tends to rely on the amount of substitutional Bi atoms, and the lower c_p_ value implies that a lower thermal energy is necessary for melting Bi-IST. Therefore, at the reset operation, the thermal energy provided by Joule heating can be reduced, which is very important for operating PRAM with lower power consumption because the power consumption for PRAM operation is strongly governed by the energy needed to shift to the amorphous phase.

**Figure 5:**
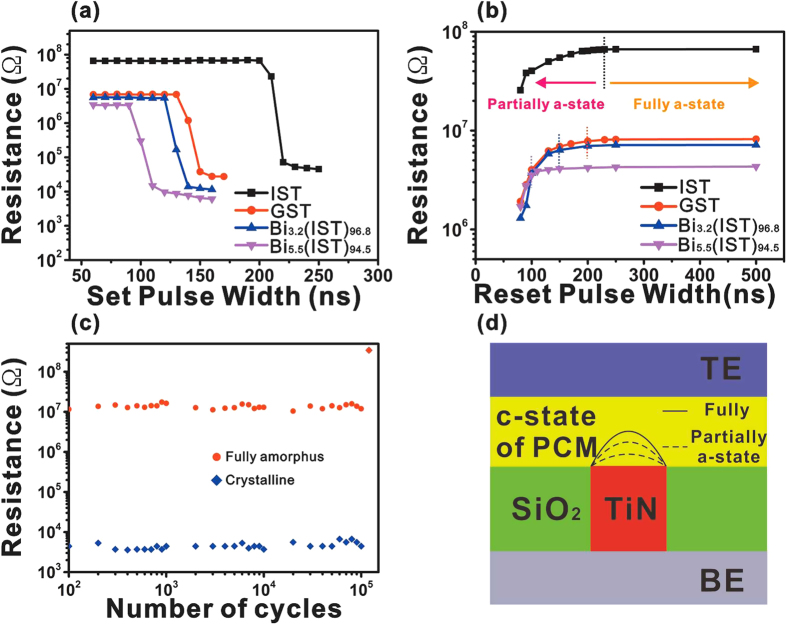
Electrical characteristics of the PRAM cell devices fabricated with IST, Bi-IST (3.2 and 5.5 at.% Bi), and GST: (**a**) set switching speed and (**b**) reset switching speed. (**c**) Writing/erasing endurance of the PRAM cell device fabricated with 5.5 at.% Bi-IST.

One of the main aims in this work is to investigate the effect of Bi atoms on switching speed of PRAM cell devices. Figure 5 shows the set and reset speeds of the devices fabricated with GST, IST, and the two different Bi-IST thin films. The set operation voltage is 2 V, and the pulse widths are varied from 80 to 240 ns in 10 ns intervals. The reset bias is 5 V, and the pulse widths are varied from 80 to 500 ns in 10 ns intervals. Figure 5(a) indicates that, for IST, set switching occurs at the pulse width of 220 ns, which is slower than the 140 ns pulse width for GST. However, as the Bi content increased from 3.2 to 5.5 at.%, the set time reduces from 140 to 100 ns. The switching speed of devices fabricated with Bi (5.5 at. %)-IST is the fastest among the considered devices. Moreover, the resistance values of Bi-IST at the set and reset states are also drastically reduced to be as low as that for GST. Ratio of heat capacity of IST, Bi(3.2&5.5at.%)-IST nearly corresponds to the ratio of operation speed. In other words, operation speed depends on the heat capacity of materials. In this work, by adding the Bi atoms into IST, the crystal structure of IST is slightly distorted, and consequentially, multiple-phase transformations occur through easy atomic migration in the distorted lattice structure. The time required to switch from the amorphous to crystalline phase is also reduced by the increasing Bi content.

The melting process speed during the reset cycle should be investigated. As indicated in Fig. 5(b), the melt-quenching process moves from the partially amorphous to the fully amorphous state during the reset pulse width. Therefore, if the reset speed is defined with the fully amorphous state, the melt-quenching process of Bi-IST is also faster than that of IST and GST. The reset time is reduced from 230 to 100 ns as the Bi content is increased from 0 to 5.5 at.%. Figure 5 also shows the relationship between the resistance changes and the set/reset speeds. The resistance of the amorphous and crystalline Bi-IST alloys (3.2 and 5.5 at.% of Bi) are relatively smaller than that of GST and IST, and the lowest resistance occurs in the case of 5.5 at.% Bi-IST. These reset characteristics imply that, during the melt quenching process, it is relatively hard to change crystalline IST seems into the amorphous state, compared with metastable-structured GST. However, by adding Bi atoms, the reset speed is also obviously faster than that of IST and GST. The PRAM cell devices are successfully operated for more than 1E5 cycles of writing/erasing pulses without resistance drift and degradation, as shown in Fig. 5(c).

**Figure 6:**
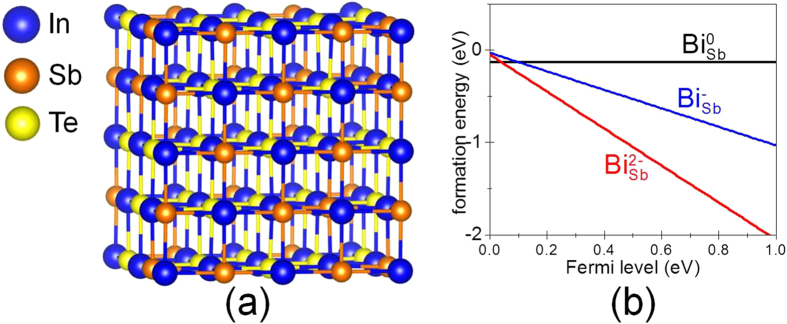
(**a**) The atomic model of IST supercell containing 64 atoms for a computational approach, (**b**) calculated formation energies of Bi dopant.

We performed DFT^22^,^23^,^24^ calculations to investigate the energetic stability of Bi-doping, indium point defect, and lattice distortions in atomic scale view. To obtain the energetic stability of Bi-doping in IST, the system total energy calculations with Bi-dopant at different sites, In, Sb, and Te sites, were performed. The IST supercell containing 64 atoms was used with the composition ratio of In_32_Sb_12_Te_20_ as presented in Fig. 6(a). Before we calculate the Bi-doping formation energies, we first found energetically stable IST supercell. The DFT calculation actually gives to the enthalpy change (*ΔH*). The calculated *ΔH* of IST supercell model is lowest when In, Sb, and Te atoms are mixed so that the same element atoms are not in the nearest sites. Assuming that the *ΔS* in *ΔG* = *ΔH*–*TΔS* will be increased by the dispersion of atoms in supercell (random mixing instead of same elements clustering), we expect that *ΔG* will be further reduced when random mixing occurs in non-zero absolute temperature. Representative test examples are in Fig. S1 of supplementary information. When one of the In, Sb, or Te atomic sites is replaced by Bi, the heat of enthalpy (*ΔH*) is obtained for the substitution using the equation


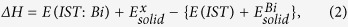


where *E(IST:Bi)* is the system total energy calculated under the condition that each of the host sites is replaced by Bi atoms in rotation, and *E(IST)* is the same total energy calculated with the host sites themselves. Moreover, 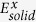 is the chemical potential of the host atoms, *x* alternates with In, Sb, and Te atoms, and 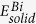 is the chemical potential of the Bi atom.

The results of the DFT calculation showing the change in *ΔH* and the maximum value of the angular distortion Δθ_max_ are summarized in Table 1 for four cases: crystalline IST (no Bi atom) and IST with a Bi atoms replacing Te, In, and Sb. Table 1 clearly suggests that Sb is the preferred substitutional site among the In, Sb, and Te host atoms and that the possible maximum value of angular distortion due to the Bi atom is 2.5°. The value of *ΔH* by Bi-doping is negative only at Sb site, −0.13 eV, which indicates that the Bi will be doped at Sb site with a good stability. The maximum angular distortion due to the inclusion of 5.5 at.% Bi in IST was 2.06°, which is fairly close to the theoretical DFT value of Δθ_max_, 2.5°. Therefore, the Bi atoms conclusively replace Sb atoms, resulting in lattice distortion because the atomic radius of Bi is slightly larger than that of Sb even though they occupy the same group in the periodic table. This distorted lattice structure may influence the phase transformation and the thermal properties. Our carrier measurements revealed that *hole* is the majority carrier with concentration of 4.25 × 10^20^/cm^3^. Bi-doping at Sb is also predicted to generate two hole carrier additionally via the reaction, Bi(solid) + Sb_Sb_ –> Bi_Sb_^2−^ + 2h^+^ (Fig. 6(b)) since Bi at Sb site tends to be mostly doubly charged (Bi_Sb_^2−^). Therefore, the reduced electrical resistance by increased Bi content is due to the increased hole carrier concentration in the *p*-type IST.

The relationship between the thermal properties and switching speeds of IST and GST indicate that the switching speed of IST is much slower than that of GST, although IST is more thermally stable than GST. Therefore, this work is aimed at exploring how to improve the switching speed of IST and at understanding the relationship between the thermal properties and the switching speed. The HRTEM images suggest that the phase-changing characteristics of IST can be improved by distorting the lattice structure slightly with substitutional Bi atoms, which replace Sb sites. Thus, Bi-IST has a slightly distorted NaCl structure, and the distortion angle is well matched with the possible maximum angle obtained with using first-principles calculations. Consequently, as the Bi content is increased, the crystallization and melting temperatures are reduced, resulting in lower activation energy and minimum enthalpy. Moreover, the specific heat of Bi-IST is also smaller than that of IST. These results reveal that, by incorporating Bi atoms, the energy barrier can be reduced for the phase changes from amorphous to crystalline and back because the distorted lattice offers migration sites for the phase transformation. Previous reports indicated that the distorted lattice induces a number of vacancies, causing faster phase transitions, and the thermal conductivity is also reduced by scattering and carrying phonons^25^,^26^,^27^. However, the activation energy of Bi-IST is slightly higher than that of GST, which means that Bi-IST has better thermal immunity to resistance drift and more stable operation than GST. The set/reset speeds of the PRAM cell devices fabricated with Bi-IST are obviously improved, and in particular, the power consumption is expected to be lower, which is very important for practical PRAM applications.

## Methods

The GST and IST thin films are sputtered on SiO_2_/Si substrates using GST and IST targets, respectively. The Bi-IST thin films are co-sputtered with IST and Bi targets. The sputtering processes are performed in an Ar atmosphere (5 m Torr). The Bi content and the sample thickness are controlled with the RF power and deposition time, and the Bi contents are varied from 0 to 5.5 at.%. All device structures consist of TiN (bottom electrode)/thin film of phase change material (programming volume)/TiN/Ti (top electrode). The contact size is 250 nm × 250 nm, and the thickness of phase change material is 100 nm. PRAM cell devices fabricated with GST, IST, and Bi-IST (Bi contents of 3.2 and 5.5 at.%) are investigated. The properties were measured with both of Keithley 4200-SCS semiconductor characterization system, Keithley 4225-PMU ultra-fast I–V module, Keithley 4225-RPM remote amplifier/switch, and Keithley 3402 pulse/pattern generator. To confirm set and reset speeds, we analyzed set speed after in the conditions of various RESET pulses. To confirm the set and reset speeds, different set and reset programming circuits are applied under the conditions of various pulse peaks, timings, and durations. Crystallization and melting temperatures of GST, IST and Bi-IST are measured in an argon atmosphere using DSC. The heating rate changes from 5 to 25 °C/min. TEM analysis is conducted with bright-field TEM (BFTEM) images, high-resolution electron microscopy (HRTEM), and FFT using FEI TITAN at 300 kV. The samples were annealed by rapid thermal annealing process at 400 °C for 30 min, followed by prepared by mechanical polishing. The diffraction patterns are also obtained according to the crystallization temperatures using XRD. The real atomic lattice structures are compared with energy stability analysis of density functional theory to predict how the Bi atoms are incorporated into the IST. For DFT^28^ calculations using Vienna Ab initio Simulation Package (VASP) code^29^, the plane-wave basis set was expanded to a cutoff energy of 450.00 eV. The average dimension of fully relaxed IST supercell with 64 atoms was 12.540 Å. The 2 × 2 × 2 k-point grids generated by the Monkhorst-Pack scheme^24^ and the projector-augmented waves (PAW) and the generalized gradient approximation (GGA) were used^30^,^31^,^32^.

## Additional Information

**How to cite this article**: Choi, M. *et al.* Lattice Distortion in In_3_SbTe_2_ Phase Change Material with Substitutional Bi. *Sci. Rep.* **5**, 12867; doi: 10.1038/srep12867 (2015).

## Supplementary Material

Supplementary Information

## Figures and Tables

**Table 1:** The calculated values of ΔH and Δθ_max_ according to the sites substituted by the Bi atom.

Substitution site	In	Sb	Te
ΔH (eV/atom)	+0.08	−0.13	+2.65
Δθ_max_ (°)	1.4	2.5	2.1
