# Corrigendum: Lattice Distortion in In_3_SbTe_2_ Phase Change Material with Substitutional Bi

**DOI:** 10.1038/srep14398

**Published:** 2015-10-01

**Authors:** Minho Choi, Heechae Choi, Seungchul Kim, Jinho Ahn, Yong Tae Kim

Scientific Reports 5: Article number: 1286710.1038/srep12867; published online: 08112015; updated: 10012015

This Article contains a typographical error in the grant number in the Acknowledgements section.

“This work is supported by Samsung Electronics, and the Ministry of Trade, Industry and Energy under the contract for the MLC-PRAM project [10039200, Development of High Performance Phase Change Materials], the Basic Science Research Program through the National Research Foundation of Korea (NRF) funded by the Korean government (MSIP) (Grant No. 2011-0028570), and KIST internal projects (Grant No. 2E25372 & 2E373)”.

should read:

“This work is supported by Samsung Electronics, and the Ministry of Trade, Industry and Energy under the contract for the MLC-PRAM project [10039200, Development of High Performance Phase Change Materials], the Basic Science Research Program through the National Research Foundation of Korea (NRF) funded by the Korean government (MSIP) (Grant No. 2011-0028570), and KIST internal projects (Grant No. 2E25372 & 2E25373)”.

