# Supplementary material for: Lattice Distortion in In3SbTe2 Phase Change Material with Substitutional Bi
Source: Sci Rep. 2015 Aug 11;5:12867. doi: 10.1038/srep12867 (PMC4531288; doi:10.1038/srep12867)
Supplement: Supplementary Information [file srep12867-s1.doc]

Lattice Distortion in In_3_SbTe_2_ Phase Change Material with Substitutional Bi

Minho Choi^1^, Heechae Choi^2^, Seungchul Kim^2^, Jinho Ahn^1*^ & Yong Tae Kim^3*^

^1^ Hanyang University, Department of Materials Science and Engineering, Seoul 133-791, Korea

^2^ Korea Institute of Science and Technology, Center for Computational Science, Seoul 136-791, Korea

^3^ Korea Institute of Science and Technology, Semiconductor Materials and Device Laboratory, Seoul 136-791, Korea

*Corresponding authors: J.A. ([jhahn@hanyang.ac.kr](mailto:jhahn@hanyang.ac.kr)), and Y.T.K. ([ytkim@kist.re.kr](mailto:ytkim@kist.re.kr))


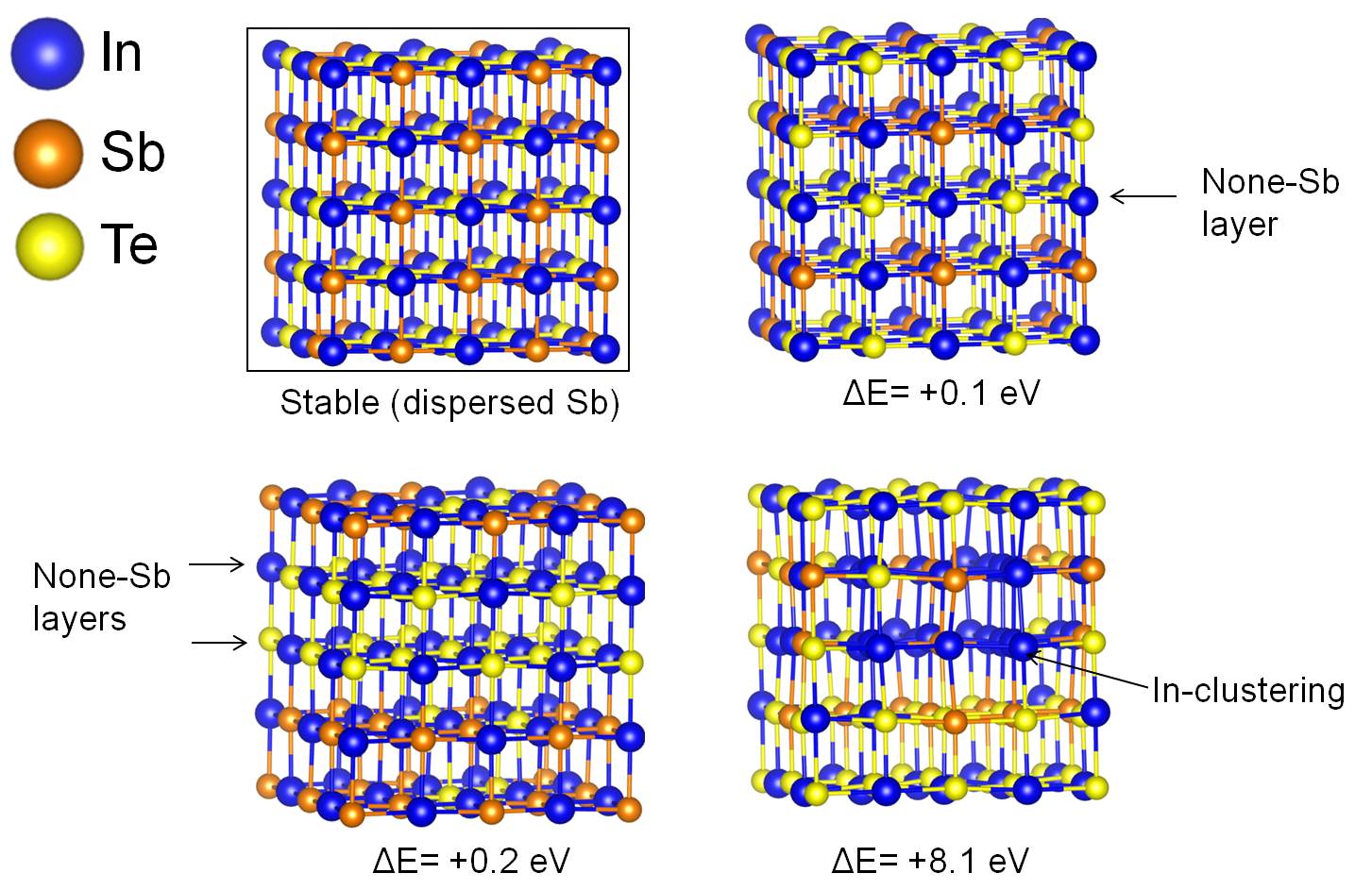


Figure S1. Building the IST and IST:Bi supercells for DFT calculations started from the Rock-salt structure of In and Te, where Sb atoms are substituted for Te afterwards. Sb atoms tend to be dispersed in further distances than clustering in near sites, as shown by the energy difference between the two models below. When Sb atom is absent in one and two layers, the system total energy were increased by 0.1 and 0.2 eV. In addition, when indium atoms clustered in the nearest distances, then the system total energy was largely increased (8.1 eV for 12 In atoms in the first nearest distance).
